# Exogenous Polyamines Influence In Vitro Microbial Adhesion to Human Mucus According to the Age of Mucus Donor

**DOI:** 10.3390/microorganisms9061239

**Published:** 2021-06-07

**Authors:** Anastasia Mantziari, Enni Mannila, Maria Carmen Collado, Seppo Salminen, Carlos Gómez-Gallego

**Affiliations:** 1 Functional Foods Forum, Faculty of Medicine, University of Turku, 20520 Turku, Finland; ensoma@utu.fi (E.M.); mcolam@iata.csic.es (M.C.C.); sepsal@utu.fi (S.S.); 2 Institute of Agrochemistry and Food Technology, National Research Council (IATA-CSIC), 46980 Valencia, Spain; 3 Institute of Public Health and Clinical Nutrition, School of Medicine, University of Eastern Finland, 70211 Kuopio, Finland

**Keywords:** *Bifidobacterium*, *Lacticaseibacillus rhamnosus*, *Cronobacter*, putrescine, spermidine, spermine, polyamines

## Abstract

Adhesion to intestinal mucus is the first step for microbiota colonization in early life. Polyamines are polycations with important physiological functions in both procaryotic and eucaryotic cells. However, their role in intestinal mucus adhesion is not known. The objective of the present study was to evaluate whether exogenous polyamines (putrescine, spermidine, spermine, and their combination) would alter the adhesive properties of *Lacticaseibacillus rhamnosus* GG (LGG), *Bifidobacterium animalis* subs. *lactis* Bb12, *Cronobacter sakazakii*, and *Escherichia coli*. Human intestinal mucus was isolated from healthy infants (0–6-month-old and 6–12-month-old) and healthy adults (25–52 years old). Spermidine significantly increased Bb12 adhesion (*p* < 0.05) in the mucus of infants (0–6 months) but reduced the adhesion of LGG in adult mucus (*p* < 0.05) with no significant effect in any of the infant groups. Spermine was more effective than polyamine combinations in reducing *C. sakazakii* (*p* < 0.05) adhesion in early infant mucus (0–6 months). The adhesion ability of *E. coli* remained unaffected by exogenous polyamines at any age in the concentrations tested. Our data suggest that polyamines may modulate the bacterial adhesion to mucus depending on the bacterial strain and depending at what age the mucus has been generated.

## 1. Introduction

Polyamines (PAs) are primordial polycations mainly known for their essential role in cell proliferation and differentiation [1]. In the human organism, putrescine (PUT), spermidine (SPD), and spermine (SPM) are the most common PAs. These compounds are required and accumulated in large amounts in rapidly growing tissues like the gastrointestinal epithelium and may provide protection against environmental challenges [2,3]. Evidence from animal studies suggests that dietary PAs may play an important role in neonatal and infant development [4,5]. Indeed, administration of exogenous PAs in suckling rats appeared to protect against decreased small intestinal mucosal weight induced by a PA deficient diet [6]. This may indicate the implication of PAs in the gastrointestinal tract maturation. In addition, PA concentration is significantly higher in preterm human milk compared to term milk while recent studies on neonatal blood metabolome revealed a negative correlation between PAs and gestational age [1,7,8]. The difference in PA concentration in preterm milk could not only reflect the different nutritional requirements of these infants according to their gestational age but also increase the integrity of the intestinal barrier as the intestinal permeability is higher in preterm infants when compared to term infants [9]. Other polyamine functions include processes such as metabolite biosynthesis and development of the immune function during the postnatal period [1,10]. In addition, PAs due to their positive charge bind to proteins and by changing their conformation may modulate signaling pathways [11].

The main sources of PAs are diet, and synthesis by the host cells or the gut microbiota [12]. More specifically, the gut microbiota is the main contributor of PAs in the lower parts of the intestine, and it is able to synthesize polyamines by multiple pathways [13,14]. Interestingly, administration of probiotics was shown to modulate and increase the PA-producing colonic microbiota [15]. Although the metabolism and function of PAs in eukaryotic cells have been widely studied [16], their effect on commensal intestinal bacteria has not been fully explored. Similar to eukaryotic cells, PUT, SPD, and SPM are the main PAs together with cadaverine, with recent studies highlighting their role in bacterial activity [17]. More specifically, PAs can influence bacteria–host interactions with various reports describing the mechanism by which PAs may contribute to bacterial pathogenesis [18,19]. For instance, spermidine production by *Pseudomonas aeruginosa* protects the pathogen against antibiotics while agmatine (a precursor of SPD and PUT) derived by the same pathogen can reduce the pro-inflammatory response of airway epithelial cells [20,21]. At the same time, polyamines are involved in pathogen biofilm synthesis and maturation [22] and their transport inside the bacterial cell contributes to pneumococcal virulence [23]. Polyamines might contribute also to the activity of beneficial microbes since polyamine transport has been evidenced in several species [24], while their production by intestinal microbiota could beneficially impact the host [25].

The role of polyamines in bacterial adherence to intestinal tract mucus and epithelium is not known but studies in protozoan parasites suggest that polyamine metabolism and secreted PUT are linked to host cell adherence [26]. We hypothesized that exogenous polyamines could modulate the adhesion of bacteria to intestinal mucus in vitro. Adhesion to intestinal mucus is the first event in the process by which intestinal microbes interact with the intestinal barrier and it could play a critical role during colonization in early life [27]. At the same time, human milk, the optimal source of nutrition for the newborn infant, is rich in polyamines. While polyamine concentration in human milk varies between mothers and is greatly affected by gestational age, the average amounts are 0.058, 0.580, and 0.825ppm for PUT, SPD, and SPM [8,28]. Similarly, Gallego and coworkers reported a median concentration of 0.019, 0.512, and 1.03 ppm for PUT, SPD, and SPM, respectively in human milk [29]. The objective of the present study was to evaluate whether exogenous PAs in similar proportions to those reported in human milk would alter the adhesive properties of microorganisms. Such property would be of importance in early life. Reference was made to well-documented probiotics *Lacticaseibacillus rhamnosus* GG and *Bifidobacterium animalis* subs. *lactis* Bb12, and the pathogen *Cronobacter sakazakii* associated with mortality in infants, and *Escherichia coli* TG1 as representative of commensal bacteria with potential pathogenic members.

## 2. Materials and Methods

### 2.1. Bacterial Strains

Four bacterial strains were used: *Lacticaseibacillus rhamnosus* GG (LGG) (ATCC 53013), *Bifidobacterium animalis* subsp. *lactis* Bb12 (DSM15954) (Chr. Hansen, Copenhagen, Denmark), *Cronobacter sakazakii* (ATCC 29544), and *Escherichia coli* TG1 (obtained from C. K. Lim). The strains were reactivated from stocks stored at −70 °C in 25% glycerol and metabolically radiolabeled with 10 μL of [3H]-thymidine (NET355001MC, Perkin Elmer, Waltham, MA, USA) that was added to each medium. LGG and *E. coli* were grown aerobically in de Man, Rogosa and Sharpe (MRS; Merck, Darmstadt, Germany) broth at 37 °C for 18–20 h while Bb12 and *C. sakazakii* were grown in Gifu Anaerobic Medium (GAM; Nissui Seiyaku Co., Tokyo, Japan) broth for 48 h at 37 °C under anaerobic conditions (10% H_2_, 10% CO_2_, and 80% N_2_; Concept 400 anaerobic chamber, Ruskinn Technology, Leeds, United Kingdom). The bacterial suspensions were centrifuged (2000× *g*, 5 min), washed twice with and resuspended in HEPES (N-2-hydroxyethylpiperazine-N′-2-ethanesulphonic acid)-Hanks buffer (HH; 10 mM of HEPES; pH 7.4). The preparations were then diluted to achieve an optical density (OD600nm) of 0.25 ± 0.01 to standardize the bacterial concentration (~10^7^–10^8^ cells/mL).

### 2.2. Isolation of Human Intestinal Mucus

Mucus was isolated from human fecal samples as described earlier [30]. The fecal samples were obtained from two age groups of healthy Finnish infants (0–6-month-old, *n* = 5; 6–12-month-old, *n* = 5) and healthy adult subjects from Finland (25–52 years old, *n* = 14) as controls. In short, fecal extracts were prepared by suspending 2 g from each sample in phosphate buffered saline (PBS; pH 7.2; 10 mM phosphate) containing 0.5 g/L to prevent bacterial growth, 1 mM phenylmethylsulfonyl fluoride (to inhibit serin proteases), 2 mM iodoacetamide (to inhibit cystein containing enzymes), 10 mM EDTA (to inhibit metalloproteases), and 0.5 g/L sodium azide (to prevent bacterial growth) followed by centrifugation at 13.800× *g* at 4 °C for 30 min. The crude mucus was then extracted by dual ethanol precipitation and further purified by size exclusion chromatography applying it to a Sepharose CL-4B column. The void volume is the first peak of the elution profile, containing high molecular weight glycoproteins with a size of molecular weight > 20 × 10^6^ DA and includes mucins [31,32]. The void volume fractions were pooled together, dialyzed, and lyophilized. For each group, equal amounts of lyophilized mucus from each individual were pooled to make a stock suspension of 10 mg/mL in HH.

### 2.3. Polyamine Preparations

Putrescine (PUT, D13208; Aldrich, Steinheim, Germany), spermidine (SPD, 2626; Sigma, Steinheim, Germany), spermine (SPM, 85590, Fluka, Steinheim, Germany), and their combination in high (MixHigh) and low (MixLow) concentrations were assessed for their effect on the adhesive properties of the above-mentioned bacteria. The polyamine stock solution was prepared by diluting each polyamine in distilled water. The final concentration in the bacterial suspension tested for each polyamine separately was 0.25 ppm. For the low concentration group, the final concentrations in the bacterial suspension were 0.025 ppm PUT, 0.5 ppm SPD, and 1 ppm SPM, and for the high concentration group 0.05 ppm PUT, 1 ppm SPD, and 2 ppm SPM. For both of the polyamine combination groups, we chose to use a proportion similar to the one reported in breast milk [29]. Polyamines were added to the radiolabeled bacterial suspension while adjusting the OD600 and just before the adhesion assay to avoid potential degradation by polyamine oxidase activity.

### 2.4. In Vitro Adhesion Assay

Intestinal mucus (0.5 mg/mL) from each age group was immobilized overnight on a polystyrene microtiter plate (Maxisorp, Nunc, Denmark). Prior to the adhesion assay, the wells were washed twice with 200 μL of HH to remove excess mucus. To study the adhesion efficacy of the tested bacteria, 100 μL of each radioactively labeled strain were mixed with polyamines, added to the polystyrene microtiter plate covered with mucus, and incubated at 37 °C for 1.5 h. To remove any unattached bacteria, the wells were washed three times with 200 μL of HH followed by a lysis step (1% SDS in 0.1 M NaOH) at 60 °C for 1 h to release the adhered bacteria. The lysate was then mixed with the scintillation liquid (Ultima Gold™ XR; Perkin Elmer, Waltham, USA) to measure the radioactivity with a 1450 Microbeta Liquid Scintillation Counter (Wallac Oy, Turku, Finland).

### 2.5. Statistical Analysis

The adhesion assay was carried out as three independent experiments, with each experiment performed in triplicate (*n* = 9). The adhesion ratio (in %) was calculated by dividing the radioactivity of the probiotics bound by the radioactivity of the probiotics added to the intestinal mucus. The adhesion ratios to the different matrixes for each bacterium affected by the presence or absence of PAs were compared by a one-way analysis of variance. Normal distribution of data was tested using the Shapiro–Wilk test. Due to normal distribution, one-way ANOVA followed by Tukey’s post hoc test was performed to analyze the differences between means. Statistics with a value of *p* < 0.05 were considered significant. All statistical analyses were carried out using the IBM SPSS statistics 23.0 software (IBM Corp., Armonk, NY, USA).

## 3. Results

**Figure 1:**
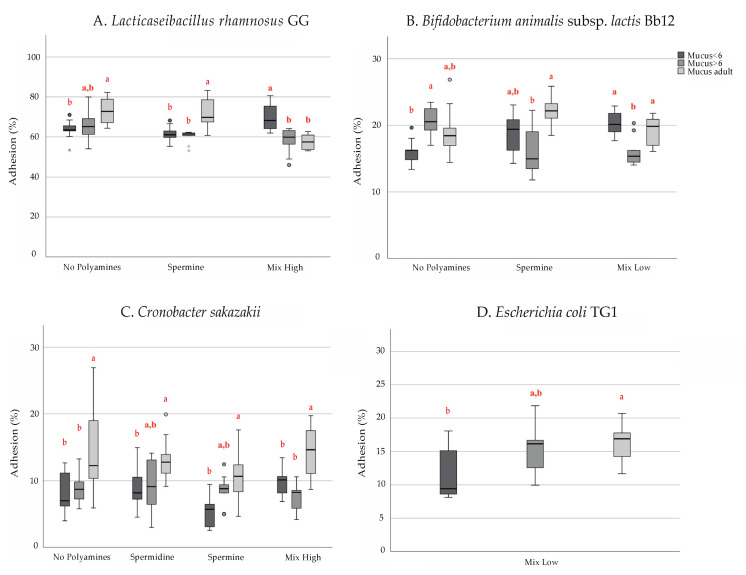
Age-dependent adhesion properties of (**A**) *Lacticaseibacillus rhamnosus* GG, (**B**) *Bifidobacterium animalis* subs. *lactis* Bb12, (**C**) *Cronobacter sakazakii*, and (**D**) *Escherichia coli* TG1 in the presence of exogenous polyamines. Box plots represent the median and quartiles of adhesion percentage. The letters are indicating significant differences at *p* < 0.05 when comparing mucus from different age categories.

**Figure 2:**
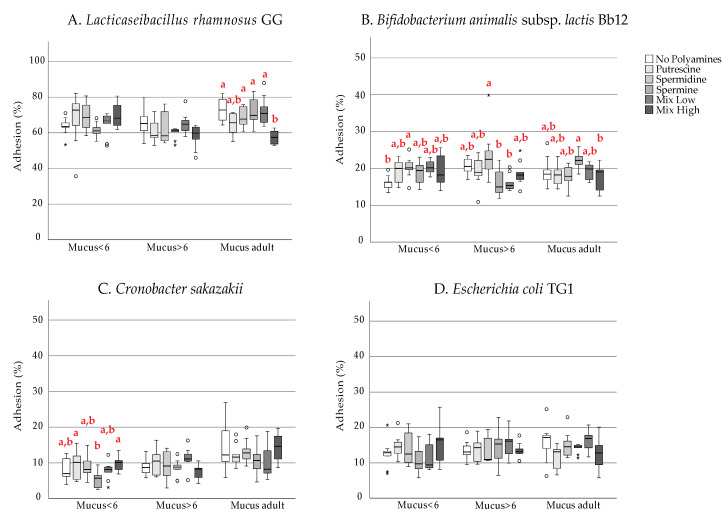
Adhesion to human intestinal mucus of (**A**) *Lacticaseibacillus rhamnosus* GG, (**B**) *Bifidobacterium animalis* subs. *lactis* Bb12, (**C**) *Cronobacter sakazakii,* and (**D**) *Escherichia coli* TG1 in the presence of exogenous polyamines. Box plots represent the median and quartiles of adhesion percentage. Different letters indicate statistically significant differences at *p* < 0.05 among polyamine exposure for each type of mucus.

Overall, in the absence of polyamines LGG, Bb12 and *C. sakazakii* present higher adherence to the adult mucus than to the infant ones (Figure 1). Both study probiotic strains presented similar adhesive capacity to our mucus model as described in earlier studies [27,33], with lower adherence for the mucus of infants in the age of 0–6 months. However, in the presence of PAs, the adherence patterns change (Figure 1) in a dependent manner regarding the type of polyamines and their concentration (Figure 2).

In the adult mucus, LGG adhesion was decreased significantly by the high PAs combination (No PAs: 73.5%, high PAs: 57.7%; *p* < 0.05; Figure 2A). The different PAs did not have a significant impact on LGG adhesion in infant mucus. Despite that, PUT and SPD (No PAs: 63.5%, PUT: 67.5%, SPD: 68.9%) appeared to slightly promote the adhesion of LGG in the younger age group, although not significantly at the concentration tested. However, this should be confirmed in future studies with higher concentrations of PAs because the concentration of PAs in the gastrointestinal tract is generally higher than in breast milk [34]. Bb12 exhibited better adhesion properties to the mucus of the older infant group (20.8%) than to the mucus originating from the feces of infants less than 6 months of age (16.1%, *p* < 0.05; Figure 1B). Additionally, Bb12 was able to adhere significantly better in mucus from the youngest group of infants in the presence of SPD (No PAs: 16.1%, SPD: 20.3%; *p* = 0.05) and well in low PAs combination (No PAs: 16.1%, low PAs: 20.2%; *p* = 0.055; Figure 2B).

Regarding *C. sakazakii*, our results show that adhesion to intestinal mucus of adults was higher than to infant mucus (0–6-month-old: 8%, 6–12-month-old: 8.9%, adult: 14.9%; Figure 1C). In addition, in the presence of SPM, the adhesion to early infant mucus is even smaller (No PAs: 8%, SPM: 5.4%; *p* < 0.05, Figure 2C). We observed that the adhesion of *E. coli* TG1 was moderate (10–20%) compared with previously reported for pathogenic *E. coli* NCTC 8603 [35,36] and was independent of the presence of exogenous polyamines at any age in the concentrations tested. Despite the non-significant effect, worth mentioning is that SPM tended to slightly decrease *E. coli* adhesion to the mucus isolated from the younger age group (No PAs: 12.5%, SPM: 11%; Figure 2D) while in the presence of low concentration of polyamines, *E. coli* adhesion was significantly lower in the early infant mucus (11.5%) than in adult mucus (16.1%, *p* < 0.05, Figure 1D).

## 4. Discussion

The different patterns in the adhesion that we observed might be due to each age group’s distinct diet. Diet is an important factor affecting the glycosylation of intestinal glycoproteins, and it has been demonstrated that the introduction of other foods besides breastfeeding during weaning could shift from high sialylation to high fucosylation terminal in mucins [37,38]. In addition, weaning was shown to increase mucus degradation and, therefore, its composition, possibly due to faster colonization of the infant gut by mucus degrading microbiota [39]. Although we did not characterize the mucus, we postulate that due to the aforementioned reasons, the mucus composition between the age groups differs. As PAs are polycations, their presence at different concentrations can alter mucus secondary structure and viscosity in a way that is directly related to the number of charges [40]. The amount of positive charges is also different for PUT (two), SPD (three), and SPM (four) [41] and it could explain some of the differences observed among them and in their mixes. In addition, different glycosylation patterns related to age and diet may influence how polyamines influence secondary mucus structure and, therefore, bacterial adhesion. However, this remains speculative and should be confirmed in future studies. Our findings regarding Bb12 suggest that exogenous polyamines could influence *Bifidobacterium* adhesion properties in early life, and dietary polyamines via breast milk or infant formula can be used to promote a healthy colonization pattern in early life. This could be an important factor to consider when assessing the stepwise colonization of the gastrointestinal tract after delivery.

Regarding *C. sakazakii*, our results are in agreement with a previously published work [42]. Most notably, the adhesion of *C. sakazakii* to early infant mucus when SPM was present was decreased, although not significantly, which may have a protective effect against *C. sakazakii* infections. Premature infants with low birth weights are a vulnerable population and are more likely to get sick by this pathogen. However, in a study by Collado and colleagues, it was shown that in the presence of specific probiotics and their combination, the adhesion of the *C. sakazakii* was reduced [42]. Consequently, future studies could focus on how PAs or their combination together with specific probiotics influence the attachment of *C. sakazakii* to mucus from infants and neonates born prematurely.

Although *E. coli* is regarded as non-mucolytic as it degrades complex carbohydrates to a small extend, several studies have demonstrated that it can interact with human mucus through its flagella [43,44]. Our findings indicate that the adhesion ability of *E. coli* remained unaffected by exogenous polyamines at any age in the concentrations tested in contrast with the rest of the bacteria used in this study. Interestingly, a previous report indicated that cadaverine, another PA with a similar structure and the same amount of positive charges as PUT, may inhibit *E. coli* adherence [45]. Based on that report, we would expect that PAs would reduce the adhesion of *E. coli* to intestinal mucus. However, the concentrations of PAs (ranging from 0.025–2 ppm) used in the current study were significantly lower than the concentration of cadaverine used by Torres and colleagues (30.7 and 51 ppm). The lack of effect could also be explained by the fact that *E. coli* uses a different PA biosynthetic pathway than the majority of human gut microbiota [13]. While *E. coli* is a minor microbial component in the adult human intestine, the genus *Escherichia* is predominant and the most abundant in the fecal microbiota composition of healthy infants at 3, 6, and 12 months of life [46,47]. At the same time, two cohort studies involving 1-month-old infants showed an association between the presence of *E. coli* in their feces with an increased risk of developing eczema [48,49]. Therefore, it would be interesting to investigate whether *E. coli* adhesion in the gastrointestinal tract can be modulated by other polyamines synthesized by intestinal microbiota—including cadaverine, agmatine, or thermospermine—and understand the mechanism behind this effect.

## 5. Conclusions

In conclusion, to our knowledge our data suggest for the first time that polyamines are able to modulate the bacterial adhesion to mucus in vitro. These properties are specific to each bacterial strain, and they are also age-specific. Our study also suggests that the concentration of exogenous polyamines needs to be considered when evaluating the adhesion properties of microorganisms. The presence of polyamines in breast milk and their relative absence in infant formulas may alter the adhesion of beneficial microorganisms such as *Bifidobacterium* in early life while hindering the adhesion of the undesirable ones. However, further confirmation of these findings is required in a more complex environment or in vivo, and also for different types and concentrations of PAs.

## Data Availability

The data that support the findings of this study are available from the corresponding author, upon request.
